# Protocol for the INterstitial lung Disease EXacerbations (INDEX) Study: a retrospective multicentre cohort study

**DOI:** 10.1136/bmjresp-2025-003949

**Published:** 2026-06-09

**Authors:** Amanda T Goodwin, Giles Dixon, Laura J White, Harvinder Virk, Iftikhar Nadeem, Gauri Saini, Fasihul Khan, Helen Parfrey, Chris J Scotton, Shaney L Barratt, Gordon Taylor, Michael Gibbons

**Affiliations:** 1 NIHR Nottingham Respiratory Biomedical Research Centre, University of Nottingham, Nottingham, UK; 2 Respiratory Medicine, University of Nottingham School of Medicine, Nottingham, UK; 3 Biodiscovery Institute, University of Nottingham, Nottingham, UK; 4 Department of Clinical & Biomedical Sciences, University of Exeter, Exeter, UK; 5 Academic Respiratory Unit, School of Health Sciences, University of Bristol, Bristol, UK; 6 Lancaster Medical School, Lancaster University, Lancaster, UK; 7 University Hospitals of Morecambe Bay NHS Foundation Trust, Kendal, UK; 8 Leicester NIHR Biomedical Research Centre, University of Leicester, Leicester, UK; 9 Princess Alexandra Hospital NHS Trust, Harlow, UK; 10 University College London Hospitals NHS Foundation Trust, London, UK; 11 Royal Papworth Hospital, Cambridge, UK; 12 University Hospitals of Leicester NHS Trust, Leicester, UK; 13 Department of Clinical & Biomedical Science, University of Exeter, Exeter, UK; 14 Bristol Interstitial Lung Disease Service, North Bristol NHS Trust, Bristol, UK; 15 Department of Health and Community Sciences, University of Exeter Faculty of Health and Life Sciences, Exeter, UK; 16 Academic Department of Respiratory Medicine, Royal Devon University Healthcare NHS Foundation Trust, Exeter, UK; 17 NIHR Exeter Biomedical Research Centre, University of Exeter, Exeter, UK

**Keywords:** Interstitial Fibrosis, Idiopathic Pulmonary Fibrosis

## Abstract

**Introduction:**

Hospitalisation for respiratory-related events is an important contributor to morbidity and mortality in individuals with fibrotic interstitial lung disease (fILD). Many such hospitalisations are due to acute exacerbations (AE-fILD), but AEs are poorly understood and there are limited evidence-based guidelines for their management. Most research in this area focuses on AEs of idiopathic pulmonary fibrosis (IPF), the commonest fILD and AEs of non-IPF fILD are less well studied. Furthermore, patients with fILD who are hospitalised with a respiratory-related event but do not meet the diagnostic criteria for AE are an important under-studied group. The INterstitial lung Disease EXacerbations (INDEX) study will describe in detail a large real-world population with fILD admitted to hospital for respiratory-related causes, including AE-fILD, as well as identify potential prognostic factors.

**Methods and analysis:**

This multicentre retrospective cohort study will analyse case notes for patients admitted to hospital between 1 September 2022 and 31 August 2023. Patient demographic data, clinical features on hospital admission, pre and postadmission investigation results, treatment approaches taken and mortality data will be collected. These data will be used to describe the patient population and to identify associations between patient, clinical presentation and treatment factors and outcomes. The primary outcome will be transplant-free survival at 90 days following the commencement of the index admission.

**Ethics and dissemination:**

The study has been approved by the Health Research Authority (HRA) (IRAS 317419) and has been prospectively registered on clinicaltrials.gov (NCT06685874). The HRA waivered the requirement for Research Ethics Committee approval due to only anonymised routinely collected clinical data being collected.

**Conclusion:**

This study will enhance our understanding of respiratory-related hospital admissions in fILD, including AE-fILD. The resulting data will describe a real-world patient population, define current standard care and may indicate prognostic and treatment factors to be assessed in future clinical trials.

**WHAT IS ALREADY KNOWN ON THIS TOPIC**

- Respiratory-related hospitalisation in fibrotic interstitial lung disease (fILD) is associated with high mortality. However, existing studies have predominantly focused on patients meeting the formal criteria for acute exacerbations, leaving a large unstudied group of patients for whom there are no clinical guidelines.

**WHAT THIS STUDY ADDS**

- INterstitial lung Disease EXacerbations (INDEX) will be a large study that provides a comprehensive analysis of respiratory-related hospitalisation in patients with fILD, including potential prognostic factors in this understudied population

**HOW THIS STUDY MIGHT AFFECT RESEARCH, PRACTICE OR POLICY**

- Evidence from INDEX may help support the generation of clinical guidelines for the management of respiratory-related hospitalisation in patients with fILD.

## Introduction

 Fibrotic interstitial lung disease (fILD) encompasses a group of conditions characterised by scarring of the lung (fibrosis) with or without inflammation. The incidence of fILDs is increasing across the world, providing a mounting disease burden.^1^,^3^ FILD can progress despite optimal treatment, a condition known as progressive pulmonary fibrosis, which has a similarly poor prognosis to idiopathic pulmonary fibrosis (IPF).^1 2 4 5^

In addition to the gradual progression typical of most fILDs, these conditions can become acutely worse, often leading to hospitalisation. A proportion of patients hospitalised with a respiratory-related event may fulfil the internationally accepted definitions of an ‘acute exacerbation’ (AE).^6^ AEs are best described in IPF, where mortality is high, at over 90% in-hospital mortality in some reports.^6^ While AEs of non-IPF fILD may share common features with AE-IPF, research on non-IPF fILD is limited, and much of the current practice in AE-fILD management is based on clinical judgement in the absence of a treatment guideline.^7^,^9^

An important yet under-studied group comprises of the patients with fILD who are hospitalised with a respiratory-related event but do not meet the full diagnostic criteria for an AE. These patients are often excluded from clinical studies, and there are numerous unanswered questions in this area, including:

- What is the typical clinical course and outcome of respiratory-related events in patients with fILD?
- Which patient factors predict the risk of in-hospital and postdischarge mortality following hospitalisation due to respiratory-related events in patients with fILD?
- How are patients with fILD who are hospitalised with an acute respiratory event managed in the real world, and are any of these approaches associated with more favourable outcomes?

To begin to address these questions, we propose the INterstitial lung Disease EXacerbations (INDEX) study, an observational study of patients with fILD and a respiratory-related hospital admission. In addition to characterising this underinvestigated patient group, these data may inform and generate hypotheses for future clinical trials in this area.

### Study aims

The primary objective of this study is to describe the patient population with fILD who are hospitalised for a respiratory-related indication in terms of patient characteristics, outcomes and usual care approaches. This study also has several secondary objectives:

1. Quantify short-term, medium and long-term transplant-free survival of patients with fILD and a respiratory-related hospital admission.
2. Define lung function trajectories following respiratory-related hospital admissions in patients with fILD.
3. Identify demographic factors, clinical presenting features and treatment approaches that are associated with mortality following respiratory-related hospital admissions in patients with fILD.
4. Describe how current National Health Service (NHS) care addresses aspects of care identified as important by patients with fILD with regards to respiratory-related hospitalisation (eg, palliative care input and oxygen therapy).

## Methods and analysis

### Study design and population

**Figure 1:**
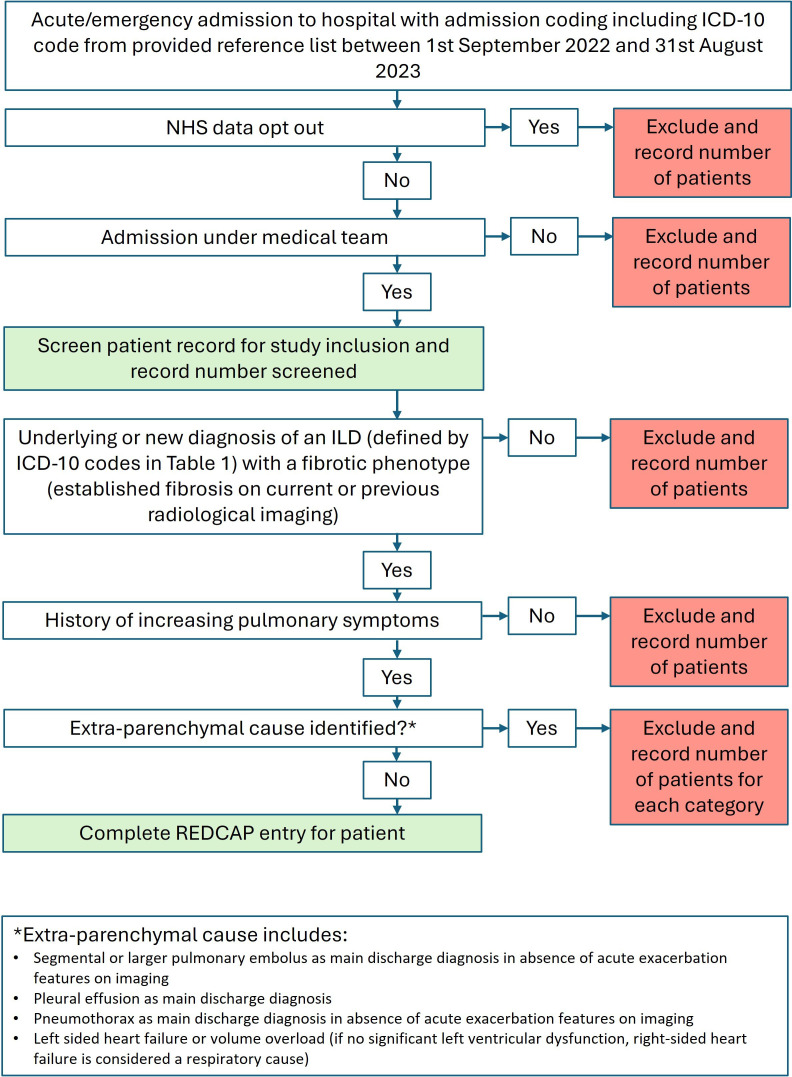
Algorithm for patient inclusion in INDEX study. Patients will be screened for inclusion using this flowchart. Diagnoses included in box indicate extra-parenchymal cause of respiratory deterioration and exclusion from INDEX study. ILD, interstitial lung disease; INDEX, INterstitial lung Disease EXacerbations; NHS, National Health Service; REDCAP, Research Electronic Data Capture.

INDEX is a multicentre retrospective observational cohort study of patients with fILD admitted to participating NHS hospital trusts with respiratory-related presentations over a 12-month period between 1 September 2022 and 31 August 2023). All patients with an admission during this study period and ICD-10 codes as indicated in table 1 will be identified, and discharge summaries reviewed against the study inclusion and exclusion criteria (figure 1). Only data from the first (index) admission during the study period will be captured. Given regional differences in clinical record keeping methods, full paper or electronic case records may be reviewed at the discretion of the local study centre.

**Table 1:** ICD-10 codes for inclusion in and exclusion from INDEX study

Category	ICD-10 code	Description
Included		
Interstitial lung disease (ILD)	J84.1	Other interstitial pulmonary diseases with fibrosis—includes diffuse pulmonary fibrosis, fibrosing alveolitis (cryptogenic), Hamman-Rich syndrome, idiopathic pulmonary fibrosis, usual interstitial pneumonia
	J84.8	Other specified interstitial pulmonary diseases
	J84.9	Interstitial pulmonary disease, unspecified
Industrial lung diseases associated with fibrosis	J60	Coal worker’s pneumoconiosis
	J61	Pneumoconiosis due to asbestos and other mineral fibres
	J62.0	Pneumoconiosis due to talc dust
	J62.8	Pneumoconiosis due to other dust containing silica
	J63.0	Aluminosis (of lung)
	J63.1	Bauxite fibrosis (of lung)
	J63.2	Berylliosis
	J63.3	Graphite fibrosis (of lung)
	J63.4	Siderosis
	J63.5	Stannosis
	J63.8	Pneumoconiosis due to other specified inorganic dusts
	J64	Unspecified pneumoconiosis
Hypersensitivity pneumonitis	J67.0	Farmer lung
	J67.1	Bagassosis
	J67.2	Bird fancier lung
	J67.3	Suberosis
	J67.4	Maltworker lung
	J67.5	Mushroom worker lung
	J67.6	Maple-bark-stripper lung
	J67.7	Air conditioner and humidifier lung
	J67.8	Hypersensitivity pneumonitis due to other organic dusts (cheese-washer lung, coffee-worker lung, fishmeal-worker lung, furrier lung, sequoiosis)
	J67.9	Hypersensitivity pneumonitis due to unspecified organic dust
Sarcoidosis	D86.0	Sarcoidosis of lung
	D86.2	Sarcoidosis of lung with sarcoidosis of lymph nodes
Lung involvement in systemic disease	M32.13	Lung involvement in SLE
	M34.01	Systemic sclerosis with lung involvement
	M35.02	Sicca syndrome with lung involvement
	M33.91	Dermatopolymyositis with lung involvement
	M33.11	Other dermatopolymyositis with lung involvement
	M33.21	Polymyositis with respiratory involvement
	M05.01	Rheumatoid lung disease with RA of unspecified site
	M05.11 - M05.17	Rheumatoid lung disease with RA of specified site
	M05.19	Rheumatoid lung disease with RA of multiple sites
Excluded		
Sarcoidosis	D86.1	Sarcoidosis of lymph nodes
Acute pneumonitis/ILD without prior evidence of fibrosis	J80.X	Adult respiratory distress syndrome
Alternative diagnoses	J81.X	Pulmonary oedema
	J82.X	Pulmonary eosinophilia, not otherwise classified

INDEX, INterstitial lung Disease EXacerbations; RA, rheumatoid arthritis; SLE, systemic lupus erythematosus.

#### Inclusion criteria

Patients will be eligible for inclusion if they meet all of the following criteria:

- Admitted to an acute NHS trust during the study period (1 September 2022–31 August 2023).
- Had an underlying or new diagnosis of an fILD (defined by ICD-10 codes in table 1), with established fibrosis on current or previous radiological imaging.
- Had increasing pulmonary symptoms on this presentation.

#### Exclusion criteria

Patients will be excluded if, at the time of hospital admission, they had:

- No underlying or new diagnosis of fILD.
- An acute presentation of pneumonitis or ILD without evidence of established fibrosis.
- A non-respiratory-related hospital admission (including elective and non-emergency admissions).
- A respiratory-related hospital admission with a primary discharge diagnosis of an extra-parenchymal cause such as:
  - Segmental or larger pulmonary embolus as main discharge diagnosis in absence of AE features on imaging.
  - Pleural effusion.
  - Pneumothorax in absence of AE features on imaging.
  - Left-sided heart failure or volume overload due to left-sided heart failure (if no significant left ventricular dysfunction, right-sided heart failure is considered a respiratory cause.
- A primary diagnosis of exacerbation of airways disease such as asthma or chronic obstructive pulmonary disease (COPD).
- Signed up to the NHS National Data Opt Out.
- An admission under non-medical specialty.

The number of patients excluded due to each criterion will be reported using a site report form.

### Subgroups

**Figure 2:**
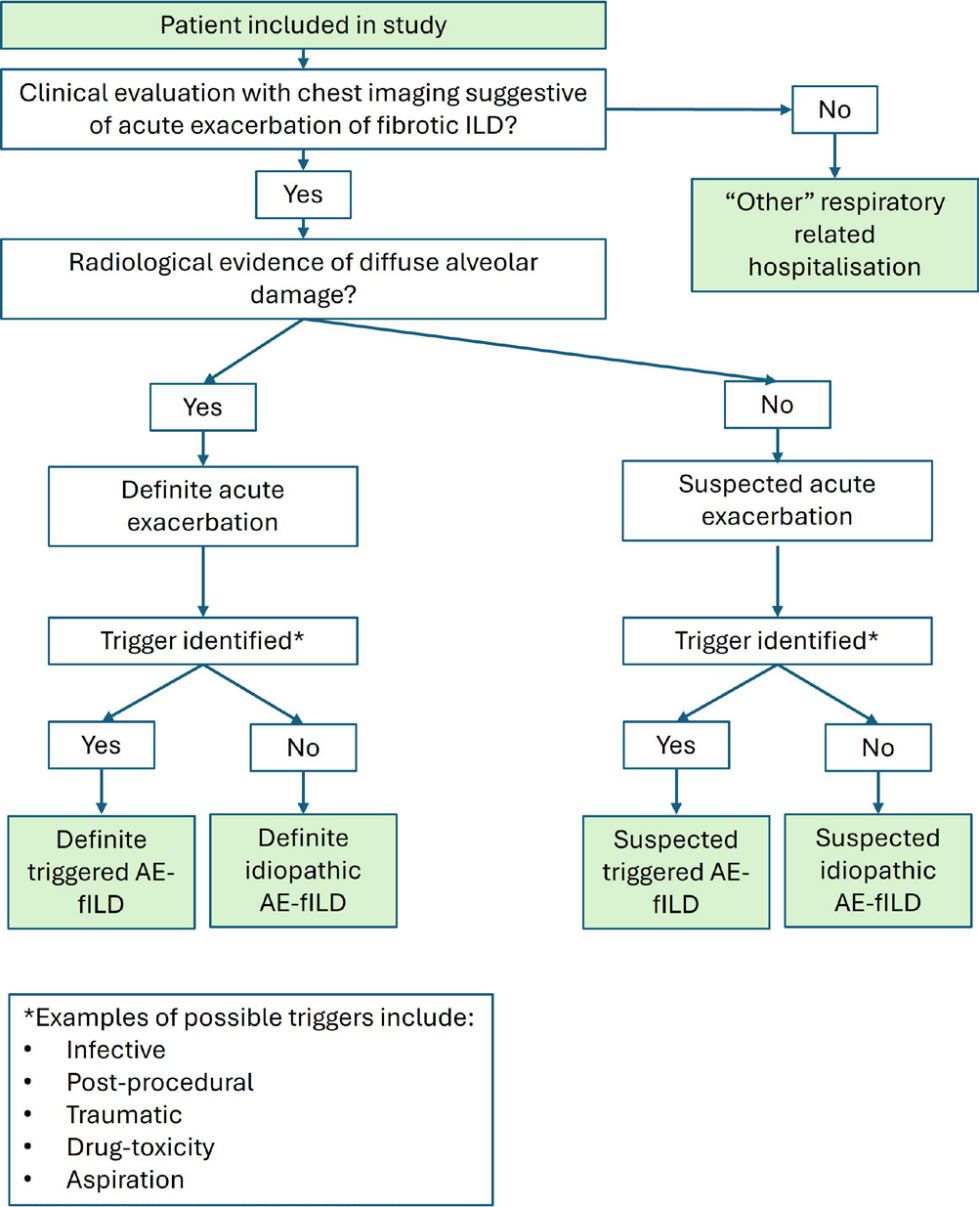
Method for assignment of study participants to subgroups. Study participants will be categorised into subgroups based on their clinical presentation. Radiological evidence of diffuse alveolar damage may include new bilateral opacities on chest X-ray, and/or new ground glass opacities on CT chest. These categories are adapted from the definitions of respiratory related hospital admissions proposed by Ford *et al*.^10^ Potential triggers for presentation are shown in the box. AE-fILD, acute exacerbation of fibrotic interstitial lung disease.

Study participants will be divided into five predefined subgroups using a method adapted from Ford *et al*^10^ (figure 2). If the overall impression of the treating clinician is that a participant has AE-fILD (according to the data available to them at the time assessment), study participants will be assigned to one of four categories. ‘Definite’ AE-fILD requires confirmed radiological signs suggestive of diffuse alveolar damage, whereas ‘suspected’ AE-fILD will not require radiological signs. Definite and suspected fILD will be further subdivided into ‘triggered’, if where a known trigger of AE, such as infection, postprocedural, trauma, aspiration or drug-toxicity, has been identified, or ‘idiopathic’ if no trigger is identified. If there is no clinical suspicion of AE, but there is a respiratory-related diagnosis, patients will be defined as ‘other’. With these pragmatic definitions, an AE is not only in keeping with the definition by Collard *et al* for AE-IPF^6^ but also allows patients who are likely to have an AE but have not had imaging to be included in the study. Furthermore, Ford *et al* showed excellent concordance between investigators and adjudication committees using this algorithm to divide respiratory-related hospital admission,^10^ making it a reproducible method between study sites.

### Endpoints

The primary endpoint of INDEX is transplant-free survival at 90 days following the commencement date of the index hospital admission. Data will also be collected on several secondary endpoints and covariates (summarised in table 2, full list of endpoints in supplementary table 1). An exploratory endpoint will also be to identify and evaluate clinical scoring systems that use routinely collected data to predict outcomes from respiratory-related hospital admission and fILD.

**Table 2:** Data to be collected on all participants included in INDEX

	Data to be collected
Primary endpoint	Transplant-free survival—90 days*
Secondary endpoints	Transplant-free survival—30 days, 6 months, 12 months*
	Change in FVC and DLCO—6 months, 1 year*
	Change in LTOT or ambulatory oxygen following discharge
	Discharge destination from index admission
Covariates: preadmission variables	Deprivation
	Demographics—sex, age, ethnicity
	ILD diagnosis—current and previous
	Body composition—height, weight
	Preadmission investigations—FVC, DLCO, echocardiogram, radiological ILD pattern
	Preadmission treatment—immunomodulation, immunosuppression, antifibrotics, oxygen
	Comorbidities—ischaemic heart disease, hypertension, COPD, diabetes, heart failure, active malignancy, bronchiectasis, anxiety, depression
Covariates: clinical presentation and management during index admission	Admission—route of presentation (eg, ED, direct admission); presenting NEWS2 score, oxygen saturations, and FiO_2_
	Treatment—oxygen; acute medications (steroids, antibiotics, diuretics, immunomodulators, symptom control); ventilatory support Type, dose, route and date(s) of administration
	Specialist input—referral and reviews (eg, respiratory, rheumatology, palliative care, HDU/ITU, tertiary centre, ECMO)
Covariates: investigations during index admission	Imaging—admission CXR, CT report(s)
	Biochemistry—WCC and differential counts, CRP, d-dimer, BNP/NT-proBNP, procalcitonin
	Microbiology—viral PCR (COVID, influenza, RSV); sputum MC&S
	Echocardiography—left/right ventricular impairment, evidence of pulmonary hypertension
Covariates: other data	ILD MDT discussion and decisions—during or within 1 year*
	Advanced care plans and ceiling of care discussions—prior to or during index admission
	Treatments on discharge—antifibrotics, immunomodulators, immunosuppressants
	Follow-up timing
	Pulmonary rehabilitation—2 years prior to or within 12 months following index admission

Full details of all data to be collected are reported in online supplemental table 1.

* From start of index admission.

BNP, brain natriuretic peptide; COPD, chronic obstructive pulmonary disease; CRP, C reactive protein; CXR, chest X-ray; DLCO, diffusing capacity for carbon monoxide; ED, emergency department; FiO_2_, fraction of inspired oxygen; FVC, forced vital capacity; ILD, interstitial lung disease; INDEX, INterstitial lung Disease EXacerbations; LTOT, long-term oxygen therapy; MC&S, microscopy, culture and sensitivity; MDT, multidisciplinary team; NEWS2, National Early Warning Score 2; NT-proBNP, N-terminal prohormone of brain natriuretic peptide; PCR, polymerase chain reaction; RSV, Respiratory Syncytial Virus; WCC, white blood cell count.

In addition to the endpoint data described above, each participating site will collect the following data to be submitted in a site report form:

- Number of patients admitted to hospital from 1 September 2022 to 31 August 2023 with 1 or more of the supplied ICD-10 codes in the coding summary.
- Number of above patients in NHS data opt out (excluded from study).
- Number of above patients admitted under non-medical specialty (excluded from study).
- Total of patients screened for study involvement.
- Total number of patients excluded (see below for exclusion criteria).
- Reasons for exclusion (numbers of patients for each).
  - Coding error, for example,
    - No underlying or new diagnosis of ILD with a fibrotic phenotype.
    - Acute presentation of pneumonitis/ILD without evidence of fibrosis.
  - Non-respiratory-related hospital admission (no increasing pulmonary symptoms).
  - Respiratory-related hospital admission due to extra-parenchymal cause.
    - Segmental or larger pulmonary embolus as main discharge diagnosis in absence of AE features on imaging.
    - Pleural effusion as main discharge diagnosis.
    - Pneumothorax as main discharge diagnosis in absence of AE features on imaging.
    - Left-sided heart failure or volume overload.

### Data collection

Data will be collected from clinical records for 12 months following the date of the index hospital admission. In cases where there are multiple eligible admissions, only the first (index) admission shall be included. Only information that is gathered and recorded as part of routine clinical care will be collected. Study centres will record participant data in an electronic case report form provided by the Sponsor via a Research Electronic Data Capture (REDCAP) online database hosted by the Sponsor (Royal Devon University Healthcare NHS Foundation Trust, Exeter, UK).

Given the nature of this study, it is expected that it may not be possible to collect all data at all sites, for example, due to variable record keeping and referral practices at individual hospital trusts. We expect that all sites will be able to report the primary outcome measure for the majority of patients. Where the administrative burden for collecting particular data points is excessive, individual sites will discuss with the trial management group (TMG) to decide whether this data can be omitted.

### Consent

The Health Research Authority waivered the requirement for patient consent due to only anonymised retrospective patient data being collected by the direct clinical team.

### Statistical analyses

The patient population will be described using appropriate summary statistics (eg, mean, median, proportions).

To assess the association between clinical and demographic variables and time to event (death), we will perform a multivariable Cox proportional hazards regression analysis. The dependent variable is the time to death, with patients who survived beyond 12 months of date of admission or were lost to follow-up being censored at the last known date or 12 months. The results of the Cox regression analysis will be expressed as HRs with 95% CIs for each variable. Kaplan-Meier survival curves will be generated for key variables to illustrate survival probabilities over time, and the log-rank test will be used to compare survival curves between groups.

To identify factors associated with survival at predefined timepoints (discharge, 90 days, 6 months and 12 months postindex admission), a multivariable logistic regression analysis will be performed. The variables to be tested will include but not be limited to age, sex, baseline lung function (eg, forced vital capacity (FVC), diffusing capacity for carbon monoxide), comorbidities, treatment regimens during the exacerbation (eg, corticosteroids, immunosuppressants) and relevant biomarkers (eg, C reactive protein levels). Where serial values are available, baseline admission values will be used. A backward stepwise selection approach will be applied to identify the most significant predictors of outcome. Variables will be removed based on statistical insignificance using a likelihood ratio test. The results of the logistic regression analysis will be reported as ORs with 95% CIs for each variable in the model.

A subgroup of patients will have repeated lung function measurements prior to and following admission to hospital. A linear mixed-effects model will be used to assess the change in the rate of FVC decline pre and postexacerbation. Dependent on the number of cases with missing data and variables associated with missing data methods for handling missing data will be undertaken with statistical support (eg, multiple imputation, complete case analysis).

### Sample size

We aim to gather data on a sample size of around 1500 patients from 20 NHS sites, including a range of tertiary referral centres and secondary care and covering a wide geographical area in the UK. The sample size is a pragmatic estimation based on pilot work (unpublished data) that suggests that a secondary care site may contribute 10–70 participants, and a tertiary care site would be expected to contribute 80–150 participants.

### Study organisation and governance

The TMG will comprise of both academic and clinical staff. The group will meet at least every 4 months and report back to the Sponsor. The TMG will also be responsible for conducting the study, maintaining the study documents, co-ordinating study publications and analysis plans and schedule.

### Patient public involvement and engagement

The study protocol was designed in partnership with people living with pulmonary fibrosis and their relatives. Patient advocate groups were consulted and had direct input into the variables selected for study and outcome measures to be investigated.

### Resident doctor research engagement

This project was developed with support from the Integrated Respiratory Research Collaborative (INSPIRE) scheme (https://www.inspirerespiratory.co.uk/). The study will invite resident doctors in training to complete the NIHR Associate Principal Investigator scheme to enable the development of research leadership skills in resident doctors.

## Discussion

Respiratory-related hospitalisation is a challenging clinical area within fILD, and research in this area is limited. While there are evidence-based guidelines for the management of fILD, these predominantly relate to the outpatient management of stable patients. There is only brief mention of a weak recommendation for steroids in AE-IPF in the international treatment guidelines, and no specific recommendations for non-IPF AE-ILD or respiratory-related events that do not fulfil the full definition of AE.^8^ It has been reported that 79% of pulmonologists with ILD expertise agree that clear guidelines for the management of AE-fILD are needed,^11^ and we propose that there is also a need for guidance for the management of hospitalised patients with respiratory-related events that do not meet the criteria for AE. However, the lack of evidence for the management of these patients has hindered the generation of such a document. Respiratory-related events in f-ILD should, therefore, be a priority area for future research.

Most existing studies in the field of respiratory events in fILD are of AE-fILD and are single or dual centre retrospective analyses that focus on one subtype of fILD.^12^,^17^ INDEX will be a large real-world evaluation of patients with a wide range of underlying fILD diagnoses, and our multicentre approach will provide a large sample size for evaluation. INDEX will provide an in-depth characterisation of patients admitted to hospital with a respiratory-related event (including AE-fILD), describe current practice in a real-world setting and give new insights into potential prognostic markers for future study. These data will generate study hypotheses for future research and may inform the development of care guidelines and treatment standards with implications for future policy and health service design.

A further strength of INDEX is the method of defining patients according to predetermined subtypes, based on the approach described by Ford *et al* for respiratory-related hospitalisation.^10^ Many of the published studies in AE-fILD define cases using criteria adapted from the International Working Group definition for AE-IPF, which requires worsening respiratory symptoms over less than 28 days, new bilateral chest infiltrates on CT imaging and no extra-pulmonary explanation for the deterioration such as heart failure or pulmonary embolism.^6^ These strict criteria may limit the number of patients eligible for study inclusion due to the requirement for CT chest imaging,^16^ and we therefore may not currently fully appreciate the significant burden of fILDs on respiratory-related hospitalisation. The approach proposed by Ford *et al* and applied to INDEX allows for the categorisation of patients into subgroups according to the evidence available and will avoid excessive exclusion of study participants to create a population representative of ‘real-world’ patients. This will allow INDEX to capture all non-extra parenchymal respiratory-related hospitalisations and, therefore, provide a broader picture of the burden of f-ILD on inpatient services.

A potential limitation of our study is the exclusion of patients with primarily extraparenchymal causes of worsening symptoms (eg, pleural effusion, pneumothorax, pulmonary embolism). While this could be an interesting subgroup to assess, we propose that these specific extraparenchymal causes of respiratory deterioration have existing treatment guidelines (eg, approaches to anticoagulation for venous thromboembolism), and underlying mechanisms that are unique to parenchymal sources of deterioration. Furthermore, the inclusion of these patients would significantly increase the data collection burden of this trainee-led study. These pathologies are therefore beyond the scope of INDEX.

A further limitation of our study is the retrospective approach, which will limit our ability to establish causative associations between exposures, such as patient risk factors and treatments given for respiratory-related events and outcomes. This is a challenging area for prospective studies due to the often high severity of the patient condition on presentation and, in the case of AEs, the relative rarity (3.19–8.38 per 100 patient years)^1417^,^19^ of these events, which make study recruitment difficult and time consuming. The retrospective approach taken in INDEX will allow us to analyse data from a large number of patients relatively quickly, providing early indicators towards aspects that warrant further study in prospective clinical trials.

Data collection will include data on comorbidities and potential risk factors and triggers for AE-fILD. Gastro-oesophageal reflux disease (GORD) is considered a risk factor for AE,^6^ but routine collection of data on GORD will not be collected in this study as it is often asymptomatic and thus inconsistently reported, which would be difficult to quantify robustly in our retrospective approach. However, we accept this as a limitation of INDEX.

INDEX will depend on clinical coding to identify potential study participants, which is a limitation as inaccurate or incomplete coding could limit patient numbers and impact our anticipated sample size. For example, we accept that patients coded as acute respiratory distress syndrome with a small burden of fibrosis may be excluded from INDEX due to the fibrotic aspect not being coded. However, we believe that this will lead to a negligible number of inappropriate exclusions from INDEX, and while it is possible that some patients who meet the inclusion criteria for INDEX are not detected by our methodology, our preliminary work suggests that we will still be able to identify a significant number of patient records for analysis.

### Ethics and dissemination

The study has been approved by the Health Research Authority (HRA) (IRAS reference number 317419) and has been prospectively registered on clinicaltrials.gov (06685874). The HRA waivered the requirement for Research Ethics Committee approval due to only anonymised routinely collected clinical data being collected.

Data from this study will be disseminated via presentations to respiratory research conferences and publications in clinical journals relevant to respiratory medicine.

## Conclusion

Respiratory-related events, leading to hospitalisation, including AE-fILD, are an important cause of morbidity in patients with fILD, but this is an understudied area. INDEX is a multicentre retrospective cohort study that describes a large fILD cohort with respiratory-related hospital admissions, including AE-fILD. These data will define current standard care, may inform future care standards and service design and could indicate prognostic and treatment factors to be assessed in future clinical trials.

## Supplementary material

10.1136/bmjresp-2025-003949 online supplemental table 1
