# Supplementary material for: Protocol for the INterstitial lung Disease EXacerbations (INDEX) Study: a retrospective multicentre cohort study
Source: BMJ Open Respir Res. 2026 Jun 9;13(1):e003949. doi: 10.1136/bmjresp-2025-003949 (PMC13264910; doi:10.1136/bmjresp-2025-003949)
Supplement: online supplemental table 1 [file bmjresp-13-1-s001.docx]

| Supplementary table: Data to be collected on all participants included in INDEX | | |
| --- | --- | --- |
|  | **Data to be collected** | **Details** |
| Primary endpoint | Transplant-free survival | At 90 days following commencement of index admission |
| Secondary endpoints | Transplant-free survival at other timepoints | At hospital discharge.  At 30 days, 6 months, and 12 months following the commencement date of index admission |
|  | Change in lung function parameters | FVC and DLCO at 6 months and 1 year following index admission |
|  | Oxygen prescriptions | Change in LTOT or ambulatory oxygen use following discharge |
|  | Discharge destination from index admission | Usual home, hospice, nursing home, residential home, community hospital, unknown |
| Covariates: Pre-admission variables | Deprivation | English Index of Multiple Deprivation Decile 2019 (England), Scottish Index Multiple Deprivation Decile 2020 (Scotland), Welsh Index of Multiple Deprivation 2019 (Wales), Northern Ireland Multiple Deprivation Measure 2017 (Northern Ireland) |
|  | Demographics | Sex, age, ethnicity |
|  | Diagnosis | Current ILD diagnosis (as per ILD MDT)  Prior ILD diagnoses (as per ILD MDT) |
|  | Body composition parameters | Height, weight |
|  | Investigations (most recent prior to index admission) | Lung function: FVC, DLCO  Echocardiogram findings  Radiological pattern of ILD |
|  | Treatment factors | ILD-specific: immunomodulation, immunosuppression, antifibrotics  Other: other immunomodulation, immunosuppression  Supportive: LTOT, ambulatory oxygen |
|  | Comorbidities | Ischaemic heart disease  Hypertension  COPD  Diabetes  Heart failure  Active malignancy  Bronchiectasis  Anxiety  Depression |
| Covariates: Clinical presentation and management during index admission | Admission details | Route of presentation (e.g. ED attendance, direct admission)  First NEWS2 score recorded  First oxygen saturations and FiO_2_ recorded |
|  | Treatment details  Type, dose, route and date(s) of administration | Oxygen prescription and target saturations  Acute medications – steroids, antibiotics, diuretics, other immunomodulators, symptom control (e.g. benzodiazepines, opiates) - Type, dose, route and date(s) of administration  Ventilatory support- CPAP, NIV, HFO_2_, IMV |
|  | Specialist team referral and reviews (dates) | Respiratory/ Rheumatology/ Palliative care teams/ ILD specialist nurse  Referral / transfer to HDU / ITU  Referral/ transfer to tertiary centre  Referral/transfer for extracorporeal membrane oxygenation (ECMO) |
| Covariates: investigations during index admission | Imaging | Admission CXR interpretation, with reference to CXR >1 month prior to admission  CT scan (s) report |
|  | Biochemistry | WCC  Neutrophil/lymphocyte and monocyte count  CRP  D-dimer  BNP/NT-proBNP  Procalcitonin |
|  | Microbiology | Viral PCR Covid/ Flu/ RSV  Sputum MC+S sent/ result |
|  | Echocardiography | Left ventricular systolic impairment  Right ventricular dilation or impairment  Echocardiographic evidence of pulmonary hypertension |
| Covariates: other data | ILD MDT discussion (timing, outcome opinion on antifibrotics) | During index admission  Within 1 year of index admission |
|  | Advanced care plans | Recorded prior to index admission  Recorded during index admission  Ceiling of care discussions |
|  | ILD treatment on discharge | antifibrotics, immunomodulators, immunosuppressants |
|  | Follow up | Date of next non-emergency hospital contact with ILD team or other relevant service |
|  | Pulmonary rehabilitation | Referrals 2 years prior to admission or in the 12 months following date of index admission |
| Abbreviations: BNP, brain natriuretic peptide; CPAP, continuous positive airway pressure; CRP, C-reactive protein; CXR, chest x ray; DLCO, diffusing capacity for carbon monoxide; ED, emergency department; FiO_2_, fraction of inspired oxygen; FVC, forced vital capacity; HFO_2_, high flow oxygen; ILD, interstitial lung disease; IMV, invasive mechanical ventilation; LTOT, long-term oxygen therapy; MC&S, microscopy, culture and sensitivity; MDT, multidisciplinary team; NEWS2, national early warning score 2; NIV, non-invasive ventilation; NT-proBNP, N-terminal prohormone of brain natriuretic peptide; PCR, polymerase chain reaction; RSV, Respiratory Syncytial Virus; WCC, white blood cell count | | |
